# Patient Outcomes Following Parasagittal Interlaminar Epidural Steroid Injections for Bilateral Lumbar Radicular Symptoms: Correlation of Contrast Spread With Symptom Relief

**DOI:** 10.7759/cureus.78817

**Published:** 2025-02-10

**Authors:** Jamal Hasoon, Omar Viswanath, Alan D Kaye, Alberto Pasqualucci, Giustino Varrassi

**Affiliations:** 1 Anesthesia, Critical Care and Pain Medicine, UTHealth, McGovern Medical School, Houston, USA; 2 Pain Management, Mountain View Headache and Spine Institute, Phoenix, USA; 3 Anesthesiology, Louisiana State University Health Sciences Center, Shreveport, USA; 4 Anesthesia and Critical Care, University of Perugia, Perugia, ITA; 5 Pain Medicine, Fondazione Paolo Procacci, Rome, ITA

**Keywords:** interlaminar epidural steroid injection, lumbar degenerative disc disease, lumbar disc herniation, lumbar radiculopathy, lumbar spondylosis, nerve root compression, parasagittal approach

## Abstract

Introduction

Bilateral lumbar radicular symptoms are commonly treated with interlaminar epidural steroid injections (ILESIs). The parasagittal approach often results in unilateral contrast spread, which may influence the degree of bilateral symptom relief. This study evaluates whether unilateral contrast spread correlates with symptom improvement in both ipsilateral and contralateral symptoms.

Methods

A retrospective review of six patients with bilateral lumbar radiculopathy secondary to lumbar degenerative disc disease, spondylosis, or disc herniation was conducted. All patients underwent ILESIs using a parasagittal approach. The injectate consisted of bupivacaine, preservative-free normal saline, and triamcinolone. Contrast spread was assessed fluoroscopically, and symptom relief was evaluated at a two-week follow-up using patient-reported outcome measures.

Results

Ipsilateral symptom relief ranged from 75% to 100% (mean: 89.2%), while contralateral relief ranged from 0% to 90% (mean: 35.8%). Notably, two patients experienced substantial bilateral relief (80-90%) despite unilateral contrast spread. These findings suggest that while ipsilateral relief is typically achieved, contralateral relief is variable.

Conclusion

The side of contrast spread strongly correlates with ipsilateral symptom improvement at short-term follow-up, while bilateral symptom relief is less predictable. Understanding the relationship between unilateral contrast spread and bilateral symptom relief is critical in optimizing injection techniques for patients with lumbar radiculopathy. Future research should focus on larger cohorts, randomized controlled trials, and direct comparisons between the parasagittal approach and midline or bilateral transforaminal techniques to optimize bilateral symptom relief strategies.

## Introduction

Bilateral lumbar radiculopathy is a painful condition often associated with nerve root compression due to degenerative changes in the lumbar spine, including lumbar degenerative disc disease, spondylosis, spinal stenosis, and disc herniation. These conditions lead to pain, numbness, or weakness radiating down both legs [1]. Epidural steroid injections (ESIs) are a common treatment for lumbar radicular pain complaints. ESIs deliver corticosteroids into the epidural space to reduce nerve root irritation and relieve pain [2,3]. This is also effective for treating cervical radiculopathy and cervical brachialgia, both as a single injection and as a continuous infusion [4-6].

There are a variety of approaches to accessing the epidural space including interlaminar, transforaminal, and caudal approaches [7-9]. The epidural space is an anatomical region located between the dura mater and the ligamentum flavum on the dorsal side. Interlaminar lumbar epidural steroid injections (ILESIs) involve delivering corticosteroids and local anesthetics into the epidural space, situated between the ligamentum flavum and the dura mater, to reduce inflammation and alleviate pain [7]. While ILESIs are widely used, different variations of this technique exist, including midline and parasagittal approaches. The parasagittal approach is commonly employed for targeted medication delivery, but it often results in unilateral contrast spread when visualized fluoroscopically [9]. This unilateral distribution raises important questions about whether the parasagittal approach can provide adequate symptom relief for patients with bilateral radicular symptoms. 

The objective of this study is to evaluate whether the side of contrast spread during parasagittal ILESIs correlates with symptom relief in patients with bilateral lumbar radiculopathy. While previous studies have evaluated the effectiveness of the parasagittal interlaminar approach for unilateral lumbar radicular symptoms, no studies have directly examined its impact on bilateral symptom relief [10-12]. Additionally, some physicians prefer using the contralateral oblique fluoroscopic view to assess needle depth, which requires a needle trajectory off the midline [13]. Understanding whether a parasagittal approach can provide meaningful relief for both ipsilateral and contralateral symptoms is clinically relevant. This study aims to assess whether unilateral contrast spread correlates with symptom improvement on both sides and to determine the potential limitations of this technique for patients with bilateral lumbar radiculopathy.

We hypothesize that although ipsilateral symptom relief will be strongly correlated with the side of contrast spread, contralateral symptom improvement may still occur to a variable degree, potentially due to systemic corticosteroid effects or delayed diffusion within the epidural space.

By identifying how contrast spread influences bilateral symptom relief, this research seeks to refine epidural injection techniques and optimize treatment strategies for patients with lumbar radiculopathy. Given the lack of studies addressing this specific issue, our findings will contribute to improving procedural decision-making and guiding future research on interventional pain management approaches. 

## Materials and methods

A retrospective review was conducted on six patients with bilateral lumbar radiculopathy, all of whom were diagnosed by board-certified pain medicine physicians based on a combination of documented pain and sensory deficits in dermatomal distributions, along with MRI findings demonstrating nerve root compression. All patients were treated at The University of Texas Health Science Center in Houston and received ILESIs performed using a parasagittal approach under fluoroscopic guidance by board-certified interventional pain physicians. All patients provided consent for treatment, and no identifying information was collected or used in this study. Patient confidentiality was strictly maintained in accordance with institutional guidelines and ethical research standards. All procedures were performed using a 20-gauge Tuohy needle, and 1.5-3 mL of iohexol contrast was injected in each patient to assess epidural contrast spread under fluoroscopic guidance.

The study aimed to assess the relationship between the side of contrast spread and clinical outcomes in bilateral symptom relief. Outcomes were assessed using the patient-reported percentage of pain relief, a standard clinical practice for evaluating response to ESIs. We utilized patient-reported percentage improvement in symptoms for each side rather than standardized pain scales such as the Visual Analog Scale (VAS) or Numerical Rating Scale (NRS). This decision was made because using objective pain scales in this context presents challenges, as patients with bilateral radicular symptoms may experience complete relief on one side but persistent pain on the other, resulting in unchanged overall pain scores. For example, a patient with a baseline pain of 10/10 who experiences 100% relief on the ipsilateral side but no relief contralaterally would still report a pain score of 10/10, despite a significant clinical improvement.

Follow-up was conducted two weeks post-procedure, which is common practice at our institution, to allow for the therapeutic effect of corticosteroids to take effect while providing a meaningful assessment of symptom relief. The two-week follow-up was chosen based on standard clinical practice, as corticosteroids typically exert their effects within this period, allowing for a standardized assessment of short-term symptom relief. While longer follow-ups would provide insight into symptom durability, many patients with persistent pain opt for repeat or alternative injections rather than delaying treatment. This study aimed to evaluate the immediate correlation between contrast spread and symptom improvement, with future research needed to assess long-term outcomes.

Inclusion criteria consisted of patients with documented bilateral lumbar radicular symptoms and imaging evidence of lumbar spine pathology contributing to nerve root compression. Exclusion criteria included previous spine surgery, unilateral symptoms, or incomplete follow-up data.

**Figure 1:**
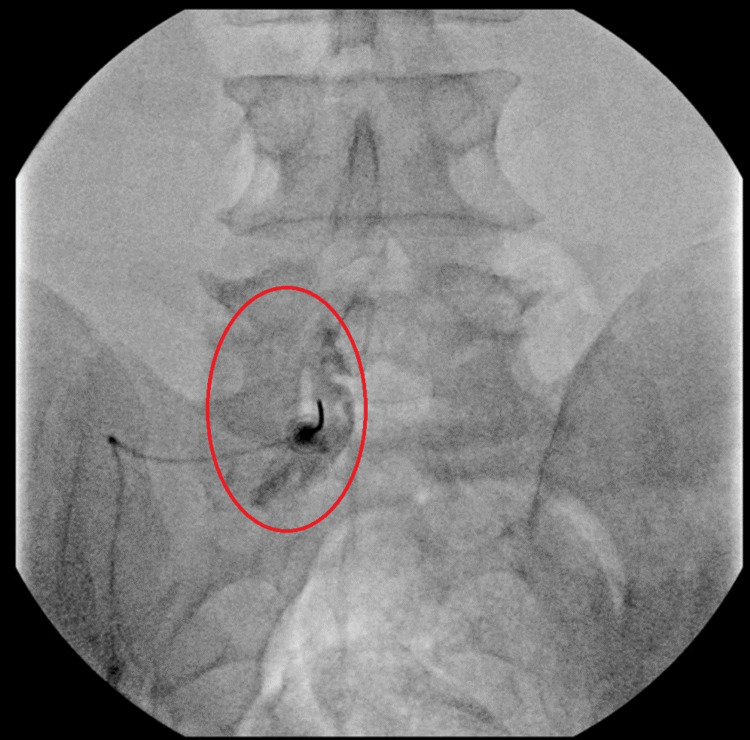
Interlaminar lumbar epidural steroid injection using a parasagittal approach Fluoroscopic image of an interlaminar lumbar epidural steroid injection at L5/S1. The red circle highlights unilateral contrast spread to the left side, with no contrast crossing the midline to the right side.

Each patient underwent an ILESI with a parasagittal approach. The patients all had unilateral contrast spread under fluoroscopic visualization with an example shown in Figure 1. All patients had an injection mixture of local anesthetic, preservative-free normal saline, and 80mg of triamcinolone.

Fluoroscopic imaging was used intraoperatively to document the side of the contrast spread (left or right). Symptom relief was evaluated at a two-week follow-up using patient-reported outcomes. The primary outcome measure was the patient-reported percentage improvement in symptoms on the ipsilateral (same side as contrast spread) and contralateral (opposite side) legs.

Descriptive statistics were used to summarize demographic and clinical data. Symptom improvement percentages were calculated and compared across ipsilateral and contralateral legs.

## Results

A total of six patients with bilateral lumbar radiculopathy were included in the study. All patients underwent ILESIs using a parasagittal approach, which resulted in unilateral contrast spread as confirmed by fluoroscopic imaging. Outcomes were assessed at a two-week follow-up, focusing on symptom improvement on the ipsilateral and contralateral sides.

Ipsilateral symptom relief ranged from 75% to 100%, with a mean of 89.2% ± 10.2%, while contralateral relief ranged from 0% to 90%, with a mean of 35.8% ± 39.5%. Individual patient outcomes varied.

Patient 1, with left-sided contrast spread at L5/S1, reported 90% bilateral symptom improvement. Patient 2, with left-sided contrast spread at L4/5, experienced 100% relief of left-sided symptoms but no improvement on the right. Patient 3, who had right-sided contrast spread at L5/S1, reported 80% bilateral improvement. Patient 4, with left-sided contrast spread at L5/S1, achieved 100% relief of left-sided symptoms but no improvement on the right. Patient 5, with left-sided contrast spread at L4/5, experienced 90% relief of left-sided symptoms and 20% improvement on the right. Patient 6, with right-sided contrast spread at L4/5, reported 75% relief on the right and 25% improvement on the left. Detailed patient demographics and injection information are summarized in Table 1.

**Table 1:** Summary of patients’ clinical conditions and outcomes DDD: Degenerative disc disease; PF: preservative free

Age	Gender	Pathology	Level of Injection	Injectate	Outcomes
56	Male	Lumbar DDD, spondylosis (L4/5, L5/S1)	L5/S1	2mL 0.25% bupivacaine, 1mL PF saline, 80mg triamcinolone (5mL total)	90% bilateral improvement
68	Female	Lumbar DDD, spondylosis, mild stenosis (L3/4, L4/5)	L4/5	1mL 0.25% bupivacaine, 1mL PF saline, 80mg triamcinolone (4mL total)	100% ipsilateral, 0% contralateral
37	Female	L5/S1 central disc protrusion	L5/S1	2mL 0.25% bupivacaine, 2mL PF saline, 80mg triamcinolone (6mL total)	80% bilateral improvement
62	Female	Lumbar DDD, spondylosis, mild stenosis (L3/4, L4/5, L5/S1)	L5/S1	1mL 0.25% bupivacaine, 2mL PF saline, 80mg triamcinolone (5mL total)	100% ipsilateral, 0% contralateral
66	Female	Lumbar DDD, spondylosis, mild stenosis (L3/4, L4/5, L5/S1)	L4/5	2mL 0.25% bupivacaine, 1mL PF saline, 80mg triamcinolone (5mL total)	90% ipsilateral, 20% contralateral
59	Male	Lumbar DDD, disc herniation (L4/5)	L4/5	2mL 0.25% bupivacaine, 2mL PF saline, 80mg triamcinolone (6mL total)	75% ipsilateral, 25% contralateral

**Figure 2:**
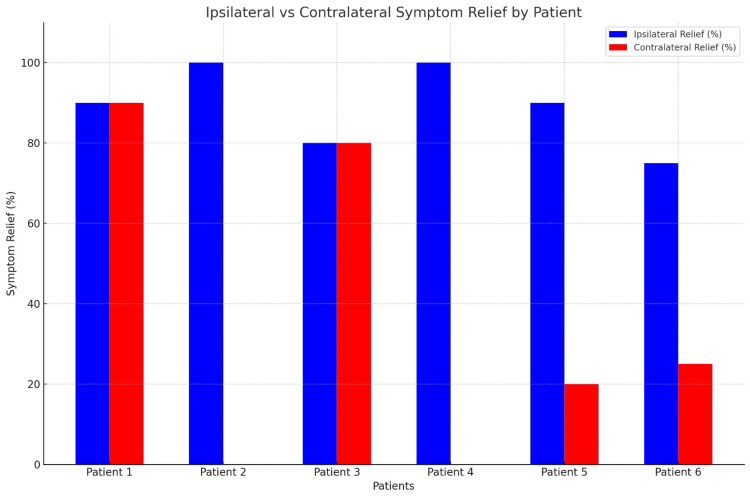
Ipsilateral vs contralateral symptom relief for the patients Blue bars represent the percentage of symptom relief on the ipsilateral side (same side as the contrast spread). Red bars represent the percentage of symptom relief on the contralateral side (opposite side from the contrast spread).

Additionally, the patient outcomes of the injections are visually represented in Figure 2.

## Discussion

The results of this study provide insights into the relationship between contrast spread during ILESIs and symptom improvement in patients with bilateral lumbar radiculopathy. Ipsilateral symptom relief was consistently high (mean 89.2% ± 10.2%), suggesting a strong correlation between the side of contrast spread and therapeutic efficacy. However, contralateral relief was highly variable (mean 35.8% ± 39.5%).

The high rates of ipsilateral relief confirm the efficacy of targeted corticosteroid and anesthetic delivery in reducing inflammation and nerve root irritation. The consistent relief on the side of contrast spread underscores the importance of accurate needle placement and fluoroscopic guidance for maximizing therapeutic outcomes.

However, contralateral symptom relief varied significantly. Two patients reported substantial improvement in both legs despite unilateral contrast spread. Possible explanations for this result may include that the injectate may spread more broadly than what is visible on fluoroscopy. Additionally, the medication may redistribute over time as the patient moves, eventually reaching the area of pathology. Another explanation could be the systemic effects of corticosteroids, which may contribute to the bilateral symptom relief observed in some patients. Additionally, baseline patient characteristics, such as severity of symptoms, underlying pathology, and comorbidities, may contribute to differences in response. Future studies with larger cohorts should evaluate these variables to determine their impact on treatment outcomes.

Previous studies have highlighted the effectiveness and often superior outcomes of parasagittal ILESIs for managing lumbosacral pain compared to alternative approaches, such as midline ILESIs and transforaminal injections [10-12]. While these findings emphasize the benefits of the parasagittal approach for targeted symptom relief, our study specifically focuses on bilateral symptom improvement. The results suggest that alternative approaches, such as midline ILESIs with bilateral contrast spread or bilateral transforaminal injections, may be more efficacious for managing patients with bilateral lumbar radiculopathy. This underscores the need for tailored treatment strategies based on the patient’s specific symptomatology and response patterns.

Patients with limited contralateral improvement may benefit from additional strategies, including repeat injections using contralateral approaches, midline ILESIs with confirmed bilateral contrast spread, or bilateral transforaminal ESIs to achieve comprehensive symptom coverage.

This study has several limitations. The small sample size restricts the generalizability of the findings and precludes robust statistical analysis. Additionally, patient-reported outcomes were assessed only at a two-week follow-up, which may not capture the full duration of therapeutic effects. The two-week follow-up was chosen because it aligns with common clinical practice for assessing the short-term efficacy of epidural steroid injections ESIs, as corticosteroids typically exert their anti-inflammatory effects within this period. This timeframe allows for a standardized comparison across patients while minimizing variability from other treatments or disease progression. However, we acknowledge that longer follow-ups would provide a more comprehensive understanding of symptom durability. Our study was intended as a preliminary investigation into the correlation between contrast spread and immediate symptom relief rather than long-term outcomes. Additionally, many patients with persistent pain after two weeks would opt for repeat or alternative injections rather than waiting for a longer assessment period. Future studies should incorporate extended follow-up intervals to evaluate the persistence of relief and guide treatment strategies. Lastly, our study design was observational and retrospective, limiting our ability to establish causality between contrast spread and symptom relief. While a strong correlation between ipsilateral relief and contrast spread was observed, contralateral relief was unpredictable. Future studies should focus on larger cohorts to validate these findings and explore the underlying mechanisms of bilateral symptom relief. Moreover, randomized controlled trials comparing unilateral versus bilateral approaches in patients with bilateral symptoms could help optimize treatment strategies. However, we believe this study provides valuable information for physicians performing ILESIs and may help guide future clinical decision-making and procedural approaches.

## Conclusions

This study demonstrates the effectiveness of ILESIs using a parasagittal approach in achieving significant short-term relief for ipsilateral symptoms. However, the small sample size limits the generalizability of these findings, and results should be interpreted with caution. The variability in contralateral relief emphasizes the importance of personalized treatment strategies and alternative procedural techniques for managing bilateral lumbar radicular symptoms, such as bilateral transforaminal ESIs or midline ILESIs with confirmed bilateral contrast spread before injection.

Our data suggest a strong correlation between ipsilateral symptom relief and contrast spread, though causality remains uncertain and requires further investigation. Given the retrospective, observational nature of this study, definitive conclusions regarding the causal relationship between contrast spread and symptom relief cannot be established. Additionally, this study evaluated symptom relief at a short-term, two-week follow-up, which may not fully capture the long-term durability of therapeutic effects or the potential for symptom recurrence. Future research should focus on larger cohorts with extended follow-up periods to assess the persistence of symptom relief and explore additional factors that may influence clinical outcomes.
